# The effect of cognitive appraisal for stressors on the oral health-related QOL of dry mouth patients

**DOI:** 10.1186/1751-0759-8-24

**Published:** 2014-10-22

**Authors:** Hirofumi Matsuoka, Itsuo Chiba, Yuji Sakano, Ichiro Saito, Yoshihiro Abiko

**Affiliations:** 1 Department of Oral Growth and Development, Division of Disease Control and Molecular Epidemiology, School of Dentistry, Health Sciences University of Hokkaido, Ishikari-Tobetsu, Hokkaido, Japan; 2 School of Psychological Science, Health Sciences University of Hokkaido, Sapporo, Hokkaido, Japan; 3 Department of Pathology, Tsurumi University School of Dental Medicine, Yokohama, Kanagawa, Japan; 4 Division of Oral Medicine and Pathology, School of Dentistry, Health Sciences University of Hokkaido, Ishikari-Tobetsu, Hokkaido, Japan

**Keywords:** Dry mouth, Cognitive appraisal, Health related QOL

## Abstract

**Background:**

Dry mouth is very common symptom, and psychological factors have an influence on this symptom. Although the influence of emotional factor related to patients with oral dryness has been examined in previous studies, the cognitive factors have not been examined thus far.

**Objective:**

The purpose of this study was to examine the influence of cognitive factors on patients with oral dryness.

**Methods:**

The participants were 106 patients complaining of oral dryness. They were required to complete a questionnaire measuring subjective oral dryness, oral-related QOL, cognition for stressors, and mood state.

**Results:**

Correlational analyses revealed that OHIP-14 is significantly related to oral dryness, appraisal for effect, appraisal for threat, and commitment. These correlations were maintained even after controlling for the influence of depression and anxiety. Using oral dryness, appraisal for effect, appraisal for threat, and commitment, cluster analysis was done and three clusters (cluster-1, severe oral dryness; cluster-2, positive cognitive style: cluster-3, negative cognitive style) were extracted. The results of ANOVA showed that the group with severe oral dryness (cluster-1) had a significantly higher score on OHIP-14 than the other two groups. There was no significant difference between the groups with positive (cluster-2) and negative (cluster-3) cognitive style.

**Conclusion:**

Although the group of patients with positive cognitive style complained of more severe oral dryness than the group with negative cognitive style, no significant difference was observed between these two groups in OHIP-14. These results indicate that cognitive factors would be a useful therapeutic target for the improvement of the oral-related QOL of patients with oral dryness.

## Introduction

Dry mouth and oral dryness are general terms that include xerostomia and hyposalivation. Xerostomia is the subjective complaint of oral dryness, and hyposalivation is the objective reduction in salivary secretion [1]. Dry mouth is a very common symptom, with an estimated incidence of between 5.5% and 40% [2,3]. The dry mouth can be caused by various factors including systemic diseases, drug effects, or radiation damage or be of psychosomatic origin [4,5]. Because salivary secretion receives autonomic innervation and sympathetic effect decreases saliva production, psychological factors such as stress, [6,7] depression, and anxiety may affect oral dryness [8-12]. Previous investigators examined if depression or anxiety affected the amount of salivary secretion [8,9,11]. Although neither depression nor anxiety directly affected the amount of salivary secretion, these psychological factors are often involved in the symptom of oral dryness [8,9,11]. The symptom of oral dryness leads to a decline in the oral health-related quality of life (QOL) [13-15]. The patient’s subjective symptoms are more closely correlated with a decrease in oral health-related QOL than with hyposalivation [16]. Hyposalivation and subjective symptoms are poorly correlated [17]. The improvement of the oral health-related QOL is important for the patients, as is the improvement of the oral symptoms. Psychological interventions for the patients may be effective for improving the oral health-related QOL.

Psychological stress models that include coping strategies for stress and cognitive styles have been applied as interventions for the psychological factors [18]. The coping strategies for the stress might be effective for suppression of the dry mouth symptoms of patients with Sjogren syndrome [19]. It is, however, still unknown how the cognitive styles affect the symptoms of patients with dry mouth. The cognitive styles could be taken advantage of as a new strategy in intervention for the psychological factors of dry mouth patients. Therefore, the present study investigated the relation between oral dryness and oral health-related QOL on the cognitive styles of patients with dry mouth.

## Materials and methods

### Participants

A total of 106 dry mouth patients (22 men and 84 women; mean age, 62.81 ± 14.38 years) were evaluated in the present study. The participants were recruited by dentists who were professionally educated about dry mouth by the Dry Mouth Society of Japan. Participants who were unable to answer the questionnaire in Japanese were excluded. The average duration of of dry mouth complaints was 46.65 months. Permission for the study was obtained from the Health Science University of Hokkaido Hospital Ethics Committee. Written consent was obtained from all patients.

### Measures

The participants answered the questionnaire in the dental clinic before receiving treatment.

### Oral dryness

In order to evaluate the degree of patient complaints of dry mouth, a question item “What is the average score of your oral dryness experienced during the past week?” was scored using an 11-point scale in which 0 represents “no dryness” and 10 represents “the dryness as bad as you can imagine”.

### Oral health-related QOL

In order to evaluate the oral health-related QOL, the Japanese version of Oral Health Impact profiles-14 (OHIP-14) [20] was employed. The OHP-14 consists of 14 items in 7 areas, including functional problems, pain, discomfort, physiological disability, psychological disability, social disability, and handicap. Each is scored on a 5-point scale where 0 represents “not at all” and 4 represents “always”. In the present study, “Because of dry mouth” was added to the beginning of each questionnaire to clarify that the complaint was due to dry mouth.

### Stressor cognition

In order to evaluate cognitions related to stressors, the Cognitive Appraisal Rating Scale (CARS) [21] was employed. The CARS consists of 4 subscales and 8 items including effect, threat, commitment, and controllability and uses a 4-point scale in which 0 represents “not at all” and 3 represents “absolutely right”. The meanings of the subscales are as follows: Effect is how much the stressor influences their lives; threat is how much they feel their stressor threatening; commitment is how much they actively engaged in resolving the stressor; control is how much they feel they can control a stressor. The subscales of CARS correspond to “challenge”, “harmful effect”, “threat”, and “controllability” as constructs advocated by Lazarus & Folkman [18].

### Mood state

In order to evaluate the mood state, the Japanese version of Profile of Mood State-Brief (POMS-B) [22] was employed. The POMS-B consists of 6 subscales and 30 items, including tension–anxiety, depression–dejection, anger–hostility, fatigue–inertia, vigor–activity, and confusion–bewilderment and uses a 5-point scale in which 0 represents “not at all” and 4 represents “extremely”.

### Statistics

Data were analyzed using SPSS version 22. Correlation analyses were performed to explore the relationship between OHIP and other variables including oral dryness and CARS. Because mood state was related to oral dryness in previous studies, [8,9,11] partial correlations were calculated between OHIP, oral dryness, and CARS to control for the effect of tension–anxiety and depression–dejection on POMS-B. Cluster analysis was conducted to clarify the subgroups of patients based on the responses on the CARS and the symptom oral dryness. To equalize the influence of variables with different scale lengths on the cluster solution, scores from CARS and the oral dryness numerical rating scale were standardized on their ranges, then used in the cluster analysis. Variables chosen for cluster modeling were selected on the basis of correlational analysis with OHIP. An agglomerative, hierarchical cluster analysis was performed with Euclidean distances used in the proximities matrix and Ward’s method used as the clustering method. Cuts made at points of large change between successive fusion levels were used to define likely cluster boundaries. To clarify the characteristics of each cluster based on oral dryness, CARS, OHIP, age and sex, one-way analysis of variance (ANOVA) and chi-square test were conducted.

## Results

The average oral dryness score was 4.59 ± 2.43 on the 11-point scale. The average OHIP-14 score was 14.33 ± 11.07. Correlation analyses were performed to explore the relationship between OHIP-14 score and each variable. The OHIP-14 score was significantly correlated with oral dryness (r = 0.57, p < 0.001), indicating that worse oral dryness probably lead to worse oral health-related QOL. The CARS controllability score showed no significant correlation with the OHIP-14 or oral dryness score. Both the OHIP-14 and oral dryness scores were positively correlated with the CARS effect, threat, and commitment (Table 1). Partial correlation analyses were performed to explore the correlations between the factors, excluding depression and anxiety. The oral dryness score was positively correlated with commitment and the OHIP-14 score was positively correlated with effect, threat, and commitment (Table 1).

**Table 1:** Correlation analyses between oral symptoms and psychological variables

	**POMS**		**CARS**			
	**Depression**	**Anxiety**	**Effect**	**Threat**	**Commitment**	**Controllability**
Subjective oral dryness	0.09 n.s.	0.21*	0.24*	0.19†	0.33***	-0.03 n.s.
			(0.15) n.s.	(0.12) n.s.	(0.27)**	(-0.20) n.s.
OHP-14	0.37***	0.48***	0.37***	0.38***	0.41***	0.03 n.s.
			(0.22)*	(0.23)*	(0.31)**	(0.08) n.s.

Number in parentheses represents the partial correlation coefficient to control the effect of tension-anxiety and depression-dejection on POMS-B.

†p < 0.10, *p < 0.05, **p < 0.01, ***p < 0.001.

Cluster analyses on effect, threat, commitment, and oral dryness revealed three clusters. The average number, age, percentage female and each variant are shown in Table 2. There were no differences in age or percentage female between the groups. ANOVA were performed for effect, threat, commitment, and oral dryness. Cluster 1 was extracted as related to sever oral dryness: The oral dryness score of cluster 1 was the highest among the clusters. Cluster 2 was extracted exhibiting positive cognitive style: Cognitive style was positive even though the oral dryness score was high. Cluster 3 was extracted as exhibiting negative cognitive style: Cognitive style was negative. The results of ANOVA for OHIP revealed that the group with severe oral dryness had a higher OHIP score than the other two groups and that there was no significant difference between the group with positive cognitive style and the group with negative cognitive style.

**Table 2:** Characteristics of the study participants based on cluster analysis

	**Severe oral dryness (cluster 1)**	**Positive cognitive style (cluster 2)**	**Negative cognitive style (cluster 3)**	**F**
n	44	42	20	
Age	63.39 (11.85)	64.20 (15.60)	58.70 (16.72)	1.04 n.s.
Female (n/%)	38/86%	32/76%	14/70%	2.63^a^ n.s.
Subjective oral dryness	6.41 (1.69)	4.01 (1.95)	1.80 (1.20)	53.23*** C1 > C2 > C3
CARS appraisal for effect	4.91 (1.48)	1.79 (1.20)	4.65 (1.42)	63.45*** C1,C3 > C2
CARS appraisal for threat	3.45 (2.06)	0.79 (1.09)	4.25 (1.97)	37.82*** C1,C3 > C2
CARS commitment	5.59 (0.66)	2.60 (1.01)	4.90 (1.41)	105.82*** C1 > C3 > C2
OHP-14	34.91 (11.75)	23.76 (6.77)	23.06 (9.43)	17.42*** C1 > C2,C3

Number in parentheses represents the standard deviation.

Comparing the three groups, each score was analysed by ANOVA, excluding the male to female (a) ratio which was analysed by chi-square test.

***p < 0.001.

## Discussion

In the present study, we examined the correlation between oral health-related QOL and the cognitive styles of dry mouth patients. The prevalence of female participants with dry mouth is much higher than that of males in the general public. The ratio of male to female participants is almost the same as in previous reports. The data may be representative of the general public [23,24]. The scores for oral health-related QOL of the three groups with dry mouth were higher than those of healthy individuals in previous reports [16,25]. The score for oral health-related QOL of the group with negative cognitive style was the same as that for the group with positive cognitive style, even though the patients in the group with a positive cognitive style complained of a lower level of oral dryness than those in the group with a negative cognitive style. This result indicates that the cognitive style of dry mouth patients may affect oral health-related QOL. The alteration of the cognitive style from negative to positive may improve the oral health-related QOL of patients with dry mouth. Approximately 40% of the patients in this study showed severe oral dryness, low oral health-related QOL, and negative cognitive style. The oral health-related QOL of these patients might be improved by the alteration of cognitive style even, if they complain of severe oral dryness.

A correlation between health-related QOL and cognitive variants, excluding depression and anxiety, was observed, indicating that the correlation was not related to the emotional factors such as depression and anxiety. These emotional factors may not be involved in the improvement of oral health-related QOL induced by the alteration of cognitive style. The previous papers mainly focused on depression and anxiety as psychological factors related to dry mouth [8,9,11]. Our study implies that some psychological factors other than depression and anxiety would be good targets for the treatment of dry mouth patients.

Commitment was correlated with the severity of oral dryness, even when depression and anxiety were excluded. The patients complained of body sensations that monopolized their attention, leading to increased sensation [26,27]. Patients with oral dryness may overly focus on the dry mouth. Distraction from the oral dryness may be an effective treatment approach.

Threat was correlated with oral health-related QOL. Patients suffering form a physical disease often have insufficient information about the disease and the patients sense a threat from the disease [28,29]. It is important to provide considerable information about the disease to the patient. The information might include that dry mouth should not be a dreaded disease, but that it is improvable.

Effect was correlated with oral health-related QOL and may be affected by both oral dryness and cognitive style. Patients who see a stressor as susceptible to effect may be less resistant to stress [30]. Patients who see the dry mouth as susceptible to effect may see a bad effect on oral health-related QOL. Cognitive behavioral therapy may useful for these patients [31-33].

The pathogenesis of dry mouth is complicated, and various factors are involved, including systemic diseases, drug effects, radiation damage, and psychosomatic origin [4,5]. Patients with systemic diseases, including diabetes, cardiovascular disease, and autoimmune diseases, often suffer from mental problems such as depression and anxiety [34-36]. The psychological factors that affect oral dryness may be involved in the symptoms of patients with systemic diseases. The pathogenesis of the dry mouth of an individual patient is too complicated to isolate accurately. Therefore, we did not evaluate individual pathogenic factors in the present study. Ideally, it would be better to know the data on each pathological condition for use in the therapeutic approach. Further investigation will be needed to clarify the data.

A limitation of this study was the study design. Because our investigation was designed as a cross-sectional study, it is difficult to draw conclusions about causal relationships between cognitive style and oral health-related QOL. Previous studies reported that psychological factors and physical illness had a mutual effect on each other [37]. Although these results imply that the cognitive style of dry mouth patients influences oral health-related QOL, longitudinal studies will be needed to explore the causal relationships between these variables.

In conclusion, the present study demonstrated that the cognitive style of dry mouth patients is correlated with their oral health-related QOL. Interventions designed to alter the cognitive style of dry mouth patients may improve their oral health-related QOL.

## Competing interests

The authors declare that they have no competing interests.

## Authors’ contributions

HM conceived of the study and drafted the manuscript. IC participated in the design of the study. YS participated making process of questionnaire. IS coordinated the enrollment of participants. YA participated in the design of the study and helped to draft the manuscript. All authors read and approved the final manuscript.
